# The impact of pharmacist oriented mode on risk control in a Chinese centralized intravenous admixture service centre

**DOI:** 10.1038/s41598-021-85077-w

**Published:** 2021-03-08

**Authors:** Hongxia Chen, Yanrong Guo, Hualing Wei, Xiaoyu Chen

**Affiliations:** 1 Department of Clinical Pharmacy, People’s Hospital of Guangxi Zhuang Autonomous Region, Nanning, 530021 Guangxi China; 2 Intravenous Admixture Services Centre, People’s Hospital of Guangxi Zhuang Autonomous Region, Nanning, 530021 Guangxi China; 3 Department of Pharmacy, People’s Hospital of Guangxi Zhuang Autonomous Region, Nanning, 530021 Guangxi China

**Keywords:** Health occupations, Risk factors

## Abstract

Centralized intravenous admixture service (CIVAS) centres, which are pharmaceutical departments found in Chinese hospitals, provide high-quality intravenous fluids and pharmaceutical services for patients, and errors in their working procedures can lead to adverse consequences. Pharmacists, the primary CIVAS centre personnel, play a role in risk control; however, to date, the effect of pharmacists’ participation in risk management has not been reported. The main aim of this study was to evaluate the pharmacist’s role in risk control and evaluate its impact. A retrospective observational study was designed to assess the principal working process in the CIVAS centre of a provincial healthcare setting. Errors in the main working process were identified, and intervention measures were formulated. The pharmacist intervention effect was evaluated by assessing the identification rate of improper prescriptions; the incidence rate of drug preparation, compounding, packaging and delivery process errors; and expenditures on wasteful drugs. There was a higher identification rate for improper prescriptions after the intervention (*P* < 0.05), while the incidence of drug preparation (*P* < 0.05), admixture (*P* < 0.05), and packaging and delivery errors (*P* < 0.01) was significantly lower; the total wasteful medication expenditure was also dramatically reduced. The potential creativity of pharmacists in error control can provide dependable intravenous drugs for patients and reduce the running expenditures for CIVAS.

## Introduction

In recent decades, advances in medical therapies have provided patients with more options than ever before; nevertheless, intravenous fluids are the primary clinical treatment due to their rapidity and effectiveness, especially in first aid medication and nutrition support. China, with its large population, consumes a large number of intravenous medicines every year^1^. Traditionally, intravenous fluids, including chemotherapy drugs, are compounded in an open environment in the ward, which may lead to contaminated intravenous fluids and the staff being exposed to toxic intravenous medication. Gradually, the Ministry of Health of China has become aware that hospital pharmacies serving hospitalized patients should routinely prepare specialized intravenous compounds in a sterile and centralized setting, and the United States Pharmacopeia (USP) Chapter <797> was consulted prior to the construction of centralized intravenous admixture service (CIVAS) centres^2^. Subsequently, the “Regulations on the Quality Management of Centralized Dispensing of Intravenous Drugs”, which provides a standardization for the establishment and administration of CIVAS centres, were enacted in 2010^3^. Currently, it is estimated that more than 1000 CIVAS centres have been established in first- or second-class Chinese hospitals; these centres have developed into an emerging pharmaceutical industry and a subdiscipline in hospital pharmacy science^1^.

Generally, Chinese CIVAS centres are characterized as multi-procedure and labour intensive, which is consistent with the fact that a large number of intravenous fluids needs to be produced from prescriptions in a limited time and that operating expenditures must be controlled within a reasonable range. To obtain the intravenous finished product, the doctor’s prescription must go through several stages, including prescription review, drug preparation and compounding, packing and delivery. Unfortunately, the multiple processes and manual operations involved are error-prone and can result in adverse events because of the reliance on human performance^4^. Worst of all, one error may result in multiple subsequent severe events. For example, unqualified chemotherapeutic fluids have been made from improper drugs or dosages, and patients can exhibit side effects or die when this fluids are administered intravenously; even if the errors are discovered before the intravenous fluid injection, fluid re-preparation will certainly lead to extra drug consumption. In addition, drug container breakage caused by careless operation is a factor in additional drug expenditure increase. Some countries have reduced errors by introducing robotics into their CIVAS centres^5^. However, this focus on the prevention of errors in the drug admixture process is not a perfect solution because it targets only a single procedure rather than the multiple, successive working processes in Chinese CIVAS centres. Furthermore, it is difficult to calculate the labour and financial costs of running automatic machines because most of the preparation and cleaning jobs have to be performed manually^6,7^. To date, risks control have not yet been addressed by effective approaches in Chinese CIVAS centres.

Meanwhile, a new round of healthcare reforms aimed at curbing the excessive and rapid growth of patient costs in Chinese public hospitals began in 2017. The minimization of patients’ pharmaceutical expenditures is a top priority for policy intervention^8,9^. One measure of policy intervention is that all governmental hospitals have an obligation to eliminate drug mark-ups and sell drugs at their purchase price^10^. Pharmacists used to make a profit for hospitals by drug sales with mark-ups, but now they are no longer the hospital revenue creator following the implementation of this policy, as a result, the remuneration for current pharmacy staff is gradually becoming a great financial burden for hospitals, although China's labour force is relatively cheaper than that of most developed countries may be a reason the majority of CIVAS centres employ labour rather than automation. Salary for pharmacists has declined more common, and pharmacists may be laid off unless they show their abilities as an innovative value generator for the hospital. On the other hand, Chinese CIVAS centres also suffer from a paradoxical dilemma. Specifically, the utilization of multiple working processes poses a significant potential for errors, and additional staff are required to recheck drugs and operations, especially in the intravenous admixture preparation and labelling phase,. Furthermore, the work of CIVAS centres is not only tiring and tedious but also fallible, and technicians easily suffer from stress and anxiety. Extra pharmacy staff members are highly beneficial for alleviating the workload and providing risk control, but staff numbers are strictly limited by hospital costs savings. Therefore, pharmacist competence should be improved to optimize working procedures and decrease risk. This would increase their value and decrease the amount of extra labour required for error management. Therefore, we performed this study to evaluate the effects of pharmacist interventions on risk control in a CIVAS centre in Guangxi.

## Methods

This study was approved by the Ethics Committee of People's Hospital of Guangxi Zhuang Autonomous Region (No. KY-ZC-2017-4). All methods were implemented in accordance with the Ethics Committees’ relevant guidelines and regulations. As this study analyzed job method data, patients did not participate in the study, and their consent was not deemed necessary by the Ethics Committee.

### Study design

The study site was the CIVAS centre of a large treatment centre in Guangxi Province. The objectives of this study were threefold. First, we sought to identify errors and related causes in the original workflow of the CIVAS centre. Second, we aimed to obtain relevant measures formulated by pharmacists to address these problems. Third, we aimed to evaluate the impact of pharmacists on risk management within the main working procedure by implementation of measures. The main working procedures from January 2018 to December 2018 and from June 2019 to May 2020 were categorized as the control group and practical group, respectively. To ensure the homogeneity of the study, the same pharmacists participated in both periods, and the entire working procedure was recorded by webcam (SONY SSC-SD36P, Sony China limited, Beijing, China) and paper documents. The rate at which improper prescriptions were identified; the incidence of errors in drug preparation, admixture, packaging and delivery processes; and the expenditures of wasteful drugs were used as assessment indexes.

### Main workflow of the control group

**Figure 1:**
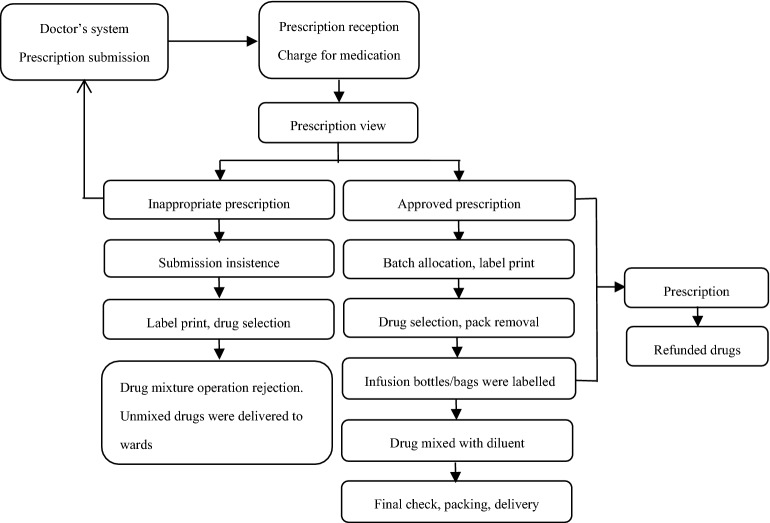
Main working process of CIVAS centres.

The workflow in our CIVAS centre is generally consistent with that of most Chinese CIVAS centres (Fig. 1). All pharmacists who are employed by the CIVAS centre have passed annual professional technique tests for their position.

#### Prescription review

Doctors’ prescriptions were transmitted to the CIVAS centre by a hospital information system, and pharmacists received prescriptions and charges for drugs. Then, the appropriateness of the prescriptions was automatically reviewed by prescription review software; unqualified prescriptions were rejected and sent back to the doctors’ system, while approved prescriptions were assigned to a batch according to the time at which the pharmacist processed the request (first batch: product delivery at 9:30 AM, second batch: product delivery at 12:00 AM, third batch: product delivery at 5:30 PM). Then, individual patient labels that included medication and batch information, as well as drug summary sheets, were printed.

#### Preprocessing step

Next, each pharmacist was assigned an equal number of patient labels and drug summary sheets, and they independently completed the following steps for these medications: all drugs were selected from the shelves following the summary sheets, the outer packages were removed, and each infusion bottle/bag was labelled with the patient’s individual information. The same drugs and their corresponding diluent were placed in the same crate. Crates colours were used to distinguish batches. Drug preparation was stopped when a prescription was cancelled due to the doctor or patient, and a full refund was permitted for prepared medicines (e.g., outer package removal, infusion bottle/bag with label) unless the medicine had been mixed with a diluent. Those prepared medicines defined as refunded drugs were put back on the shelf or assigned to other patients to whom they were prescribed. Pharmacists usually focus their attention on successive jobs; hardly anyone notices that refunded drugs have not yet been dealt with. Thus, it is important for medication in the warehouse to be well managed.

#### Admixing step

Cytotoxic fluids and parenteral nutrition fluids were produced respectively in the biosafety cabinet and laminar flow cabinet in a class 10,000 clean room. The stoppers of each drug and diluent vial/bottle that directly contacted a syringe needle were cleaned with 75% ethanol before the admixing operation. Then, each drug was mixed with the related diluent and injected into an infusion bottle/bag, after which time empty bag(s) or vial(s) were taken away and confirmed outside the clean room as part of the final verification. The workbenches, cabinets and staff gloves were cleaned with 75% ethanol following completion of the compound process of each bottle/bag. The above steps were performed independently by each pharmacist.

#### Packaging and delivery

A final check of quality and appearance was conducted by the pharmacist. Products that passed the final check were allocated to their wards by scanning the infusion bottle/bag label’s bar code and printing delivery sheets. If the rejected prescription was submitted insistently, the CIVAS centre rejected the mix of drug and diluent. The unmixed drug and label for intravenous fluids were still provided to the wards.

### Identification of workflow RISK

Workflow risk was defined as errors that occurred at any step within the main working procedures where the prescription and its related drug were physically handled or manipulated by a healthcare professional and could result in physical injury to the patient, occupational injury to the staff or financial loss to the CIVAS centre. All deviations in the working process that were potentially hazardous to patients, such as deviations in the preparation or combination of drugs from the doctors’ prescriptions, deviations from the hospitals’ intravenous administration policy, or deviations from the manufacturers’ instructions, were also included in the definition of risk. It is worth noting that medications in incorrectly compounded batches were considered a risk because the deviation in administration of finished intravenous fluids at the prescribed drug administration time may result in drug instability or deferred patient treatment. There were no definite indexes with which to evaluate or measure errors due to inadequate aseptic techniques or potential cytotoxic medication leaks; thus, errors or potential errors from these factors were not included in this study. A research panel consisted of one chief pharmacist, one associate chief pharmacist, two pharmacists trained in standardized operations of cytotoxic and parenteral drugs, and two pharmacists with more than 10 years of experience in drug management. The chief pharmacist and associate chief pharmacists reviewed all video and paper records and identified risks or potential risks independently. Acquisition differences were resolved by reviewing the workflow from the video. The identification results were summarized in a table, and each panel member was required to participate in cause analysis. The details are summarized in Table 1.

**Table 1:** Critical risks points and their main sources in the main working procedures of CIVAS centres.

Working procedure	Type of error	Main reasons	Clinical or CIVAS centre’s seriousness
Drug management	Drug beyond expiration date	1. No reasonable plans for purchasing medicine 2. Inattention to expiration date 3. “first expire, first out” principle unawareness	Therapeutic failure
	1. Drug spoilage 2. Breakage of bag(s) or vial(s) 3. Medication selection error	1. Unfamiliar with drugs’ storage requirements 2. Careless warehouse entry check or sloppy shelf transfer 3. No distinguishable labels on shelves for similar medicines (e.g., name, appearance) or fatigued inattention	1. Unpredictable serious consequences 2. Air contamination, a waste of drugs 3. Error in drug preparation and intravenous admixture procedure
Prescription review	Failure to identify incorrect prescription	1. Obsolescence of prescription review software 2. No sufficient time for prescription review 3. Overdependence on the review software	Adverse reaction(e.g., allergy, physical impairment)/therapeutic failure/unpredictable serious consequences/death
Preprocessing step	1. Medication selection error 2. Incorrect label allocation for infusion bottle/bags 3. Drug preparations were allocated to incorrect batches 4. Refunded drugs were continually prepared for original users 5. Drug container breakage	1. No distinguishable labels on shelves for similar medicines (e.g., name, appearance) or careless selection 2. Small label font lead to improper recognition or careless operation 3. Incorrect label allocation for different batches 4. Operators’ busyness and carelessness 5. Improper /fatigued operation	1–2. Incorrect medication for next step 3. Disruption of treatment plan/ therapeutic failure 4. A waste of drugs 5. Air contamination, a waste of drugs
Admixing step	1. Refunded drugs were continually produced for original users 2. Medications were mixed with diluent in incorrect batches 3. Diluent errors (wrong diluent or wrong volume usage) 4. Preparation of wrong drug dosage 5. Additive omission 6. Operation spill 7. Drug container breakage	1. Operators’ busyness and carelessness 2. Batch checks were not conducted before compounding 3–5, 7. Careless individual operation or excessive work pressure 6. Pharmacists were in a hurry to produce fluids	1. A waste of drugs 2. Disruption of treatment plan/therapeutic failure 3–4. Uncertain drug effects 5. Therapeutic failure 6–7. Air contamination, a waste of drugs
Packaging and delivery	1. Unusual changes in finished fluids (e.g., colour change, sediment, impurities, etc.) were not noticed 2. Leak-proof package was not sealed 3. Finished fluids were assigned to incorrect packages and delivered 4. Omission of finished fluids scan and delivery sheet printout	No sufficient time	1. Unpredictable serious consequences 2. Cytotoxic substances posed a threat to staff’s health 3–4. Transportation delay and therapy delay

### Main workflow of the practical group

Specific countermeasures (Table 2) had been approved by the director of our CIVAS centre and then incorporated into the original working steps. Every step was signed by the operator, and the person who made an error can be tracked once a risk is discovered. A flexible group that aimed to address two issues was established: (1) heavy workload alleviation in admixing, packing, and delivery steps helped reduce fatigued-related errors, and (2) efficient product completion ensured that patients’ therapeutic plans were not delayed (its four members come from original staff rather than additional employment).

**Table 2:** Description of pharmacists’ interventions in main errors or potential risks.

Working procedure	Pharmacists’ roles and/or interventions	Main outcomes
Drug storage & administration	Specific pharmacists were put in charge of drug examination (e.g., acceptance, expiration date, quality), shelf transfer, inventory and storage conditions management	Substandard drugs were prevented from entering the CIVAS centre. Drugs with broken containers and those beyond the expiration date were identified and disposed. The centre's medicines were well managed.
	Distinctive, alert labels and different colour medication boxes were employed for indistinguishable drugs and cytotoxic medications. Cytotoxic medications were separated from other medicines by independent shelves.	The probability of selecting an incorrect medicine was significantly reduced.
Prescription review	Pharmacists were responsible for continuously updating prescription review software.	The improved prescription review software not only kept up with therapeutic progress but also identified more improper prescriptions, which provided safe intravenous medications for patients.
	Prescription submission was strictly limited to the day before mixture completion except some special cases, which had to be submitted in a specific time window (8:00-10:30 AM, 3:00-4:30 PM)	Prescriptions were no longer submitted casually to the CIVAS centre, providing sufficient time for careful consideration.
	Pharmacists reviewed the appropriateness of prescriptions approved by automatic review software and recorded improper prescription information.	Drug-related problems were identified, which prevented the occurrence of adverse events.
	Pharmacists provided physicians with improper prescription feedback.	Feedback corrected physicians' stereotypical compromised cognition and improved their therapeutic skills.
	Pharmacists analysed prescription errors retrospectively and recommended optimized therapeutic schemes to clinicians.	The occurrence of incorrect prescriptions was significantly reduced
	Pharmacists participated in monitoring patients receiving intravenous medication	Pharmacists and physicians collaborated on the prevention and solution of incorrect medication regimen, which achieved a high acceptance rate among the patients
Preprocessing step	Medicine selection from shelves and outer package removal were carried out by pharmacists and confirmed by their working partners.	Medicine selection was more accurate and efficient
	Maximum font sizes for labels and indications of its notable points were implemented to improve readability. A recheck of the infusion bottle/bag’ agreement with the patients’ label and batch allocation was conducted while medications were prepared	This measure played a cautionary role in highlighting potential risks for subsequent steps
	Refunded drugs were addressed by the assigned pharmacists	Refunded medications have been dealt with appropriately, reducing waste due to faulty operations.
Admixing process	All medications and related operations were checked again before implementation	Refunded drugs were prevented from being produced for previous patients. Medications in the wrong batch were identified before the admixture phase.
	Each added ingredient and its dosage were rechecked and monitored by a working partner when a pharmacist was operating. Consistency with the patients’ label and the used vials was inspected for error prevention	Multiple careful examinations curbed error occurrence in this phase
	Pharmacists designed leak-proof packages for finished cytotoxic fluids	The possibility of a cytotoxic fluid leak was greatly reduced
	Extra pharmacist participated in the compounding phase, sealing of leak-proof packages of fluids, and transport of final products and used vials to the packing room	The additional technicians not only enabled fluid products to be sent to wards within the required time, but also helped to alleviate overload and identify errors
Packaging and delivery	The impurities, precipitates, seal, batch allocation of finished intravenous fluids were examined again by a pharmacist	Fluids with problems were identified
	Electronic reminders for scanning and delivery sheet omission were utilized according to the pharmacists’ requirement	The medication delivery was not delayed
	Pharmacists carried out a monthly survey of intravenous medication usage and storage in the wards	Problems were addressed in a timely manner, and physicians and nurses were generally satisfied with these approaches

#### Drug management

**Figure 2:**
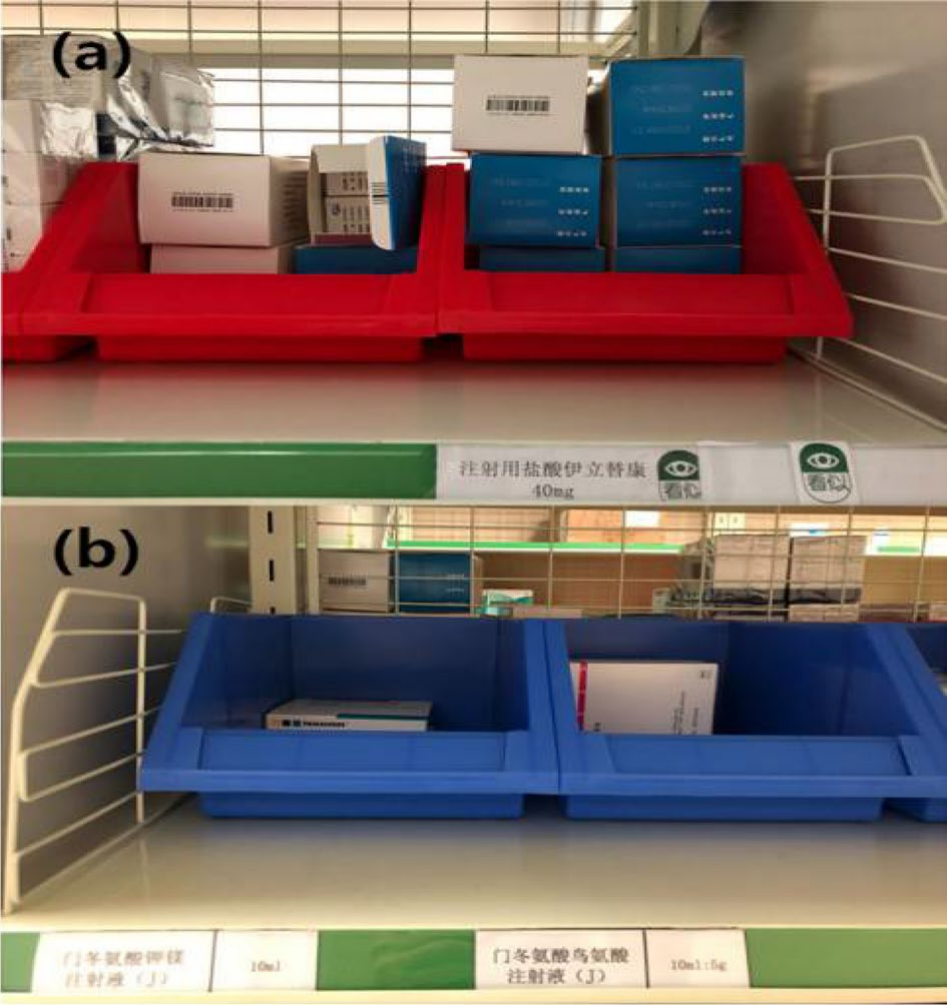
**(a)** Cytotoxic drugs are stored in red boxes, **(b)** non- cytotoxic drugs are stored in blue boxes.

**Figure 3:**
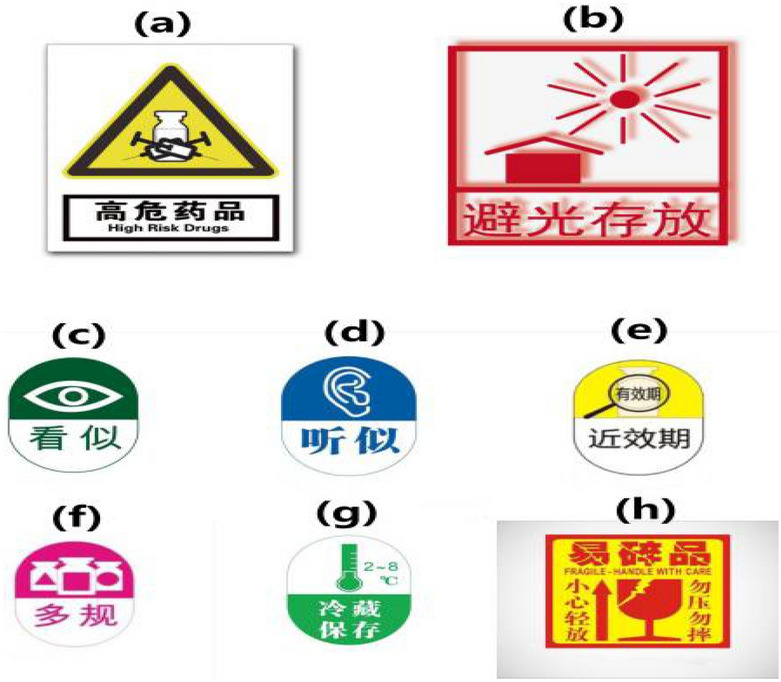
Warning sticker for **(a)** chemotherapeutical medications, **(b)** dark place storage drugs requiring dark storage, **(c)** drugs with similar external packaging drugs, **(d)** drugs with similar names, **(e)** drugs approached expiry approaching expiration date, **(f)** drugs with different contents, **(g)** drugs must store in refrigerator requiring refrigeration, **(h)** fragile medications. Assemblage of images was derived from version 9.6.45 of Hamrick VueScan (https://www.hamrick.com/).

Two permanent pharmacists were appointed to perform daily drug management each day. They were responsible for all aspects of medication monitoring, making sure that all medications were manufactured through consistent processes. Distinctive/alert boxes and various pattern label designs were an innovation of this task. Specifically, different coloured boxes (Fig. 2) were used to distinguish cytotoxic from non-cytotoxic drugs; labels (Fig. 3) with sticky material could be placed on shelves, refrigerators or other places to indicate their contents; and cytotoxic and other high-risk medications were placed on independent shelves for identification purposes.

#### Prescription review

During the prescription review process, the appropriateness and accuracy of drug employment were assessed by manually reviewing prescriptions that had been approved by the software. If inappropriate prescriptions were found in the review process, the pharmacists not only informed the prescribing physician immediately but also analysed them monthly; then, corresponding analysis results and modification suggestions were given to the physicians. Besides, the prescription submission time was limited to the day before the admixture stage for the purpose of achieving a sufficient handling time. They were received only in cases of urgent prescriptions that needed to be dealt with instantly after submission, and the case submission had to be performed within a certain time window (8:00–10:30 AM, 3:00–4:30 PM) even to have a chance of being accepted. It should be noted that the intelligent review software could not be upgraded automatically; thus, staff periodically renewed the automatic review software. The updated software included adding new legal medical evidence, such as that from drug manufacturers, and various treatment guidelines.

#### Preprocessing step

Pharmacists performed preparation tasks in teams of two members. One staff member selected medications, removed the outer packaging, and labelled the infusion bottle/bag, while a second person monitored and rechecked these operations. After drug review, this process continued with a “third” pharmacist performing a bottle/bag check, i.e., a check that the medication bottle/bag agreed with the patient’s label, as the first step towards ensuring that the correct medication had been selected. In addition, the pharmacists improved the readability of the patient labels by increasing the font size and marking key points. In terms of refunded drugs, the assigned pharmacists disposed of them to prevent them from being used for the original patients due to carelessness or busyness.

#### Admixing step

Consistency in medications and labelling were carefully examined in the cabinets before the start of an admixture. Then, a pharmacy technician filled a syringe with the required volume of drug needed for the dosage, and a second pharmacist checked the syringe before injecting it into a bag. The technician then injected the contents of the syringe under the observation of a second staff member, and the final product, along with the used vials and empty syringes, was checked by the second member. After this process, contaminated syringes and vials were disposed of in the biosafety cabinet, avoiding the risk of contaminating other areas of the clean room. An individual leak-proof package for each final cytotoxic product was introduced by the pharmacist. The flexibility group participating in this step helped provide products to the wards on time.

#### Packaging and delivery

The quality and appearance of final products, as well as delivery sheets, were examined by two pharmacists. When they struggled with a large volume of examination tasks, flexible shift staff shared the workload with them. The update scanning software that be enabled to provide an electronic reminder of missing printouts of scans and delivery orders was introduced by pharmacists. In addition, pharmacists conducted a monthly survey of intravenous fluid usage in the wards, which provided feedback for product improvement.

### Statistical analysis

Categorical variables between the two groups were analysed by the chi-square test. Differences in numerical variables between the two groups were analysed by the independent samples T test. P < 0.05 was considered significant. All tests were conducted with SPSS 22.0 software (https://www.ibm.com/cn-zh).

### Ethics approval

This study was approved by the Ethics Committee of People's Hospital of Guangxi Zhuang Autonomous Region (No. KY-ZC-2017-4).

### Consent to publish

The publication has been approved by all co-authors.

## Results

### Identification rate of improper prescriptions

Pharmacists rechecked the prescriptions that had been approved by review software, revealing a higher rate of identifying improper prescriptions in the practical group (1%) than in the control group (0.24%) (P < 0.05, Table 3).

**Table 3:** Assessment of identification rate of improper prescriptions.

Item	Control group (n = 39,798)	Practice group (n = 39,810)	Χ2 value	P value
Medications usage beyond indications	10(0.025)	48(0.12)	17.057	0.017
Combination drug therapy without evidence	23(0.058)	51(0.13)		
Wrong drug selected	8(0.020)	44(0.11)		
Wrong drug dosage	15(0.038)	46(0.12)		
Wrong diluent volume	16(0.040)	66(0.17)		
Wrong diluent	13(0.033)	51(0.13)		
Wrong administration route of medication	7(0.018)	49(0.12)		
Wrong frequency for of medication usage	2(0.005)	45(0.11)		
Total	94(0.24)	400(1.00)		

#### Incidence of preprocessing errors

The actual number of prepared drugs was lower in both groups because of cancelled prescriptions and refunded drugs. Error incidence rates were lower in the practical group (0.22%) than in the control group (0.57%) (P < 0.05, Table 4) after intervention with corresponding measures.

**Table 4:** Assessment of the error incidence in the preprocessing process.

Item	Control group (n = 39,519)	Practice group (n = 39,552)	Χ2- value	P value
Preparation of prescription inconsistency medicine	53(0.13)	12(0.03)	13.484	0.036
Preparation of unauthorized medications	19(0.05)	3(0.01)		
Preparation of unqualified medication	23(0.06)	14(0.04)		
Medication container breakage	27(0.07)	17(0.04)		
Medications with incorrect label	49(0.12)	13(0.03)		
Wrong batch allocation for medications	25(0.06)	16(0.04)		
Continuous preparation of refunded medications for a previous patient	30(0.08)	12(0.03)		
Total	226(0.57)	87(0.22)		

#### Incidence of errors in the admixing process

The number of medications in the admixing process was less than that in the preprocessing phase due to instant prescription cancellations and drug refunds. In this process, the results indicated that the error incidence in the practical group (0.07%) was lower than that in the control group (0.64%) (P < 0.05, Table 5).

**Table 5:** Assessment of the error incidence during the admixing process.

Item	Control group (n = 39,208)	Practice group (n = 39,215)	Χ2 value	P value
Diluent errors (use of incorrect diluent or incorrect volume)	62(0.16)	8(0.02)	12.836	0.025
Incorrect dosage	22(0.06)	5(0.01)		
Medications omission	40(0.10)	1(0.003)		
Undetected impurities and precipitates	28(0.07)	9(0.02)		
Medication container breakage	56(0.14)	7(0.02)		
Continuous admixture of refunded drugs from a previous patient	42(0.11)	2(0.005)		
Total	250(0.64)	29(0.07)		

#### Incidence of errors in packaging and delivery

After the measures for the packing and delivery process were implemented by technicians, the error rate in the practical group (0.14%) was significantly lower than that in the control group (0.79%) (P < 0.01, Table 6).

**Table 6:** Assessment of the error incidence during packaging and delivery.

Item	Control group (n = 39,208)	Practice group (n = 39,215)	Χ2 value	P value
Undetected impurities and precipitates	31(0.08)	12(0.03)	29.682	0.000
Fluids transportation in the incorrect batch or ward	59(0.15)	10(0.03)		
Crack in the inner packages of fluids	32(0.08)	15(0.04)		
Sealing omission for leak-proof packages of fluids	55(0.14)	6(0.02)		
Scan omission	97(0.25)	3(0.008)		
Delivery sheet omission	34(0.09)	9(0.02)		
Total	308(0.79)	55(0.14)		

#### Wasteful drug expenditures

Compared with the cost of wasted drugs before the intervention, the value after the intervention decreased dramatically (P < 0.05, Table 7). Following the pharmacists’ involvement, the total cost of wasted drugs decreased from ¥52,112.20 to ¥9,865.80.

**Table 7:** Wasteful drug expenditures (¥) before and after practice.

Stage	Control group	Practice group	Difference	Χ2	P value
Drug administration	8326.3	1877.6	6448.7	2.786	0.047
Prescription review	5433.8	1240.3	4193.5		
Preprocessing	16,780.4	2915.5	13,864.9		
Admixing	18,193.5	3092.9	15,100.6		
Packing and transportation	3378.2	739.5	2638.7		
Total	52,112.2	9865.8	42,246.4		

## Discussion

CIVAS is an approach that reduces risks and errors related to the preparation and administration of injectable drugs and protects staff from hazardous drug exposure, according to previous literature^11,12^. Although the error rate was not too high in the control group, which may be attributed to the technicians’ good training and responsibility, a basic competency for each staff member in the CIVAS centre, it was still necessary to strictly control the incidence of procedure errors, which are not only the cause of serious adverse consequences for patients, such as therapeutic failure, physical impairment, and death, but also the trigger of toxic pollution and financial loss at CIVAS centres. The pharmacist-oriented practice outlined here provides an opportunity to control those working procedure risks for patients, the CIVAS centre and its technicians. The incorporation of this practice during regular working procedures allowed its easy implementation in our CIVAS centre.

The implementation of this practice achieved a notable degree of effectiveness from the start, largely due to permanent administrators who were assigned to conduct these tasks. These technicians are familiar with medical examination and inventory management, which resulted in the attainment of several goals: unqualified products were recognized from a large number of drugs before achieving CIVAS centre acceptance, and monthly drug consumption and purchase were estimated accurately to avoid shortages or overstock.

In the control group, selection errors and item return mix-ups were likely because of the close proximity of stored medications with similar names and appearances (e.g., epirubicin and doxorubicin, different medium- and long-chain fat emulsions), the same medications with different specifications (e.g., 10 mg and 50 mg nedaplatin), or the same medications from different manufacturers (oxaliplatin from Sanofi-Aventis, France and Jiangsu Hengrui, China). These issues were resolved through the use of alert labels and different colour storage boxes as well as being placed on separate shelves in the practical group. Furthermore, assigning specific staff to handle refunded drugs helps separate them from the consecutively prepared medications and prevents them from being reassigned to the original patients.

Whereas a fully automated software is able to store manufacturer instructions and medical database information as well as automatic identify improper prescriptions, prescriptions approved by the software still have to be handled manually. It is impractical for software information systems to automatically upgrade to the latest medical knowledge, view prescriptions associated with the patient's laboratory tests, collect imaging and pathological results, or judge certain improper cases. Pharmacists aim to improve such situations through their roles in collecting state-of-the-art medication data, including guidelines, manufacturer instructions, and databases; rebuilding software information systems; taking patients’ examination results into account while reviewing prescriptions; and making decisions regarding special prescriptions based on their expertise. As our practice has shown that manual prescription viewing tasks are extremely time consuming despite being performed by experienced technicians and are highly dependent on the pharmacist’s professional level, we are confident that we could complete this task by curbing the submission of prescriptions made one day prior to the drug admixture, with the exception of certain emergency cases involving chemotherapy regimen adjustments or medications needed for emergency care, to provide sufficient time for review. Hence the increase in identifying improper prescriptions is likely attributed to prescription review software upgrades and appropriate prescription submission times.

The medication preparation and mixture process is the step that is most prone to error, and previous data have revealed that errors are greatly affected by medication administration, the use of robotic drug hybrid devices, and staff training^6,7,13^. Even if our early drug management practices reduce errors in the subsequent work procedure, electronic medication storage and inventory systems are a better complement to manual error intervention^14^. Unfortunately, most hospitals may be reluctant to invest large sums of money into electronic devices because 70.1% of the 137 CIVAS centres in China operate at a loss^3^. Consequently, we adopted a practical method in which the preprocessing and admixing of each bottle/bag of intravenous drug is strictly controlled by two pharmacists for accuracy (process cross-checks and signature label confirmation). If any human error occurred during these or subsequent working procedures, the signature could be traced back to whoever made the error. In addition, the original method for training pharmacists on optimal mixture protocols and standardization continued. In the practical group results, the lower error rate in the preparation and mixture processes may be attributed to the effectiveness of these approaches. The expenditures associated with drug waste in the practical group were lower than those in the control group. We assume that the prevention of workflow errors, such as medication container breakage and incorrect admixing procedures, reduced the quantity of wasted drugs and decreased expenditures and running costs directly^15–17^. In addition, the prevention of medication container breakage, especially for cytotoxic drugs, significantly reduced the occupational exposure of staff. Finally, higher quality and more accurate intravenous fluids were transported to the correct wards in the practical group than in the control group, suggesting the improved effect of pharmacist participation in rechecking the quality of the product and the delivery sheets.

If the same group pharmacists participated in both the control and practical groups, why were their working efficiencies and results so different? There are three possible reasons for this discrepancy. First, the traditional working procedure in the CIVAS centre lacked an effective management approach, which not only posed challenges to risk control but also resulted in a failure to appreciate and exploit the pharmacists’ abilities. Second, the pharmacists in the practical group played a role in optimizing their work steps according to correct management techniques, and the results of their involvement were welcomed by physicians, nurses, and patients, which enabled them to experience a sense of individual importance and value. The sense of achievement, and stress from healthcare reforms thus encouraged the pharmacists to create advanced working methods. Third, staff in flexible group not only are capable of relieving fatigue-triggered errors but also enable intravenous products to be completed in the required time.

This practice was created and conducted by pharmacists focused on the development of their professional skills and may not be applicable to nurse-based CIVAS centres. However, other CIVAS centres may be able to achieve similar effects by modifying these procedures.

## Conclusion

A pharmacist-focused practice during working procedures increased the improper prescription identification rate and reduced the incidence of errors in drug preprocessing, admixing, packaging, and delivery processes. This study provides practical information for CIVAS centres in most Chinese hospitals.

## Data Availability

This article is distributed under the terms of the Creative Commons Attribution 4.0 International License (http://creativecommons.org/licenses/by/4.0/), which permits unrestricted use, distribution, and reproduction in any medium, provided appropriate credit is given to the original author(s) and the source, a link to the Creative Commons license is provided, and any changes are indicated.
